# Freeze‐Dried Instant Juice Powders From Guava, Amla, and Jamun: Evaluation of Physicochemical Properties, Bioactive Compounds, and Consumer Acceptance

**DOI:** 10.1002/fsn3.70374

**Published:** 2025-06-12

**Authors:** Md. Rafiquzzaman, Md Akram Hossain, Rifat Rafique Dwip, Musfika Jahan, S. M. Kamrul Hasan

**Affiliations:** ^1^ Department of Food Processing and Preservation Faculty of Engineering, Hajee Mohammad Danesh Science and Technology University Dinajpur Bangladesh

**Keywords:** antioxidant activity, freeze‐drying, fruit juice powder, functional foods, tropical fruits, vitamin C

## Abstract

The nutritional enhancement and optimum physical properties in formulating instant fruit juice powder are challenging. This study explored the development and evaluation of freeze‐dried instant fruit juice powders from three tropical fruits: Guava ( *Psidium guajava* ), Amla ( *Phyllanthus emblica* ), and Jamun/black plum ( *Syzygium cumini* ), aimed at offering health‐promoting benefits and ensuring year‐round supply. The freeze‐drying method was chosen so that it helps in retaining the nutritional and sensory qualities of the fruits. Consequently, three types of juice powders were developed: Guava Juice Powder (GJP), Amla Juice Powder (AJP), and Jamun Juice Powder (JJP), through the mixing of dried fruit powders with other key ingredients. These juice powders were evaluated for their physical, chemical, and functional characteristics, as well as consumer appeal. The findings revealed that the powders successfully preserved the physical qualities essential for good storage and consumer use. They retained high levels of vitamin C (116.21–176.89 μM), carotenoids (5.09–8.03 μM β‐carotene), phenolics (289.56–822.62 mg GAE/100 g), flavonoids (98.21–607.74 mg QE/100 g), and antioxidant activity (43.05–45.90 μM Trolox equivalents), highlighting their potential as functional food ingredients. Sensory evaluations showed high acceptability, particularly for JJP (8.29), with GJP (7.97) and AJP (7.74) also performing well. This research contributes to the functional foods market by offering a novel method for utilizing tropical fruits, thereby expanding consumer options, supporting health, and minimizing fruit waste.

## Graphical abstract

This study developed freeze‐dried instant beverage powders from guava, amla, and jamun, aiming to enhance nutritional intake and provide year‐round availability. The powders retained high levels of vitamin C, phenolics, and flavonoids, demonstrating significant antioxidant properties. Sensory evaluations indicated a strong consumer preference for the jamun formulation, suggesting its potential for commercial success.

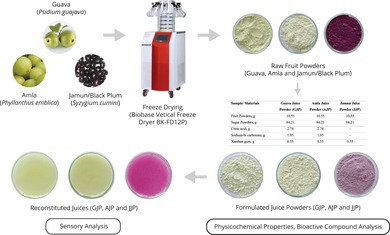

## 1 Introduction

In the current world, consumers are more aware of their food choices and actively looking for food options that are innovative and health‐promoting. The increase in demand for healthier food is driving the phenomenal growth of the functional food market, expected to reach $267 billion by 2027 (Shavronskaya et al. 2023). The rising consumer interest and market expansion have paved the way for the growing popularity of healthy juice powders, alongside other functional foods. Juice powder, with its ability to utilize diverse ingredient matrices, particularly fruits, has emerged as a prominent choice (Chaturvedi et al. 2021).

Fruits, being naturally sweet and low in calories, are an essential part of a healthier diet; they are also rich in necessary vitamins, minerals, and many other functional components (Hasan, Kabir, et al. 2022). Consequently, global fruit consumption has risen, increasing the demand for year‐round availability. However, the high moisture content and water activity inherent in fresh fruits make them highly perishable and susceptible to microbial spoilage and enzymatic degradation. This significantly limits shelf life and contributes to substantial post‐harvest losses, potentially reaching 40%–50% for fresh produce (Salehi and Aghajanzadeh 2020).

Developing fruit‐based juice powders presents a viable strategy to address these challenges. By significantly reducing moisture and water activity through drying, the shelf life of fruit‐derived products can be dramatically extended, simplifying storage, transportation, and handling (Feng et al. 2024). Furthermore, these powders align with consumer dietary trends seeking convenient, fruit‐based nutrition. During the development of functional food products from fruits, it is of paramount importance to understand the effect of processing technologies on the functional properties, the key attributes that contribute to the desirability of fruits.

Commercially, several drying technologies including sun drying, solar drying, tray drying, drum drying, and spray drying are already being used by the industry to process a variety of fruit‐based food products (Sonarthi et al. 2023). A primary challenge across these methods, especially those involving heat, is the retention of valuable, often heat‐sensitive, bioactive compounds (such as vitamins and antioxidants) and the preservation of desirable sensory qualities (like natural flavor, aroma, and color) which are crucial for consumer acceptance of nutrient‐enriched foods. Among the available techniques, freeze‐drying (lyophilization) is increasingly recognized for its superior ability to preserve food quality (Ratti 2024). Operating at low temperatures and removing water via sublimation minimizes thermal degradation and structural collapse. This makes freeze‐drying particularly advantageous for retaining bioactive compound integrity and the delicate sensory profile (flavor, aroma, color) characteristic of the original fruit compared to aforementioned conventional heat‐based methods (Aryaee et al. 2023). Reflecting its potential, numerous studies worldwide have already investigated the application of freeze‐drying for various fruits, including guava, sapota, and papaya (Athmaselvi et al. 2014), grapefruit (Agudelo et al. 2017), mango (Zotarelli et al. 2022), Jambolan and Acerola (de Matos et al. 2023), red grape, mulberry, and strawberry (Aryaee et al. 2023).

This study focuses on three tropical fruits, Guava ( *Psidium guajava* ), a popular fruit globally, that is particularly rich in ascorbic acid (vitamin C), total phenolics, total carotenoids, dietary fiber, and minerals (Kumar et al. 2022). Amla ( *Phyllanthus emblica* ), utilized as both food and medicine for centuries across Asian countries, offers numerous nutritional benefits (Zhang et al. 2024). Jamun/black plum ( *Syzygium cumini* ) is native to the Asian subcontinent and is rich in essential phytochemicals and antioxidants (Aqil et al. 2014). These fruits are well‐known for their nutritional properties and rich sensory profiles. Despite their benefits, the seasonal availability and perishability of these fruits limit their year‐round accessibility. Transforming them into instant juice powders via freeze‐drying offers a promising avenue to create novel, convenient functional food choices, ensure year‐round availability, enhance sustainability by reducing post‐harvest losses, and deliver the sensory and nutritional qualities consumers expect. Therefore, the objective of this study was to formulate instant juice powders from guava, amla, and jamun using freeze‐drying technology, and to comprehensively evaluate the resulting powders in terms of their physicochemical properties, bioactive compound content, functional characteristics, and sensory attributes to assess their quality and potential as appealing functional juice ingredients.

## 2 Materials and Methods

**FIGURE 1:**
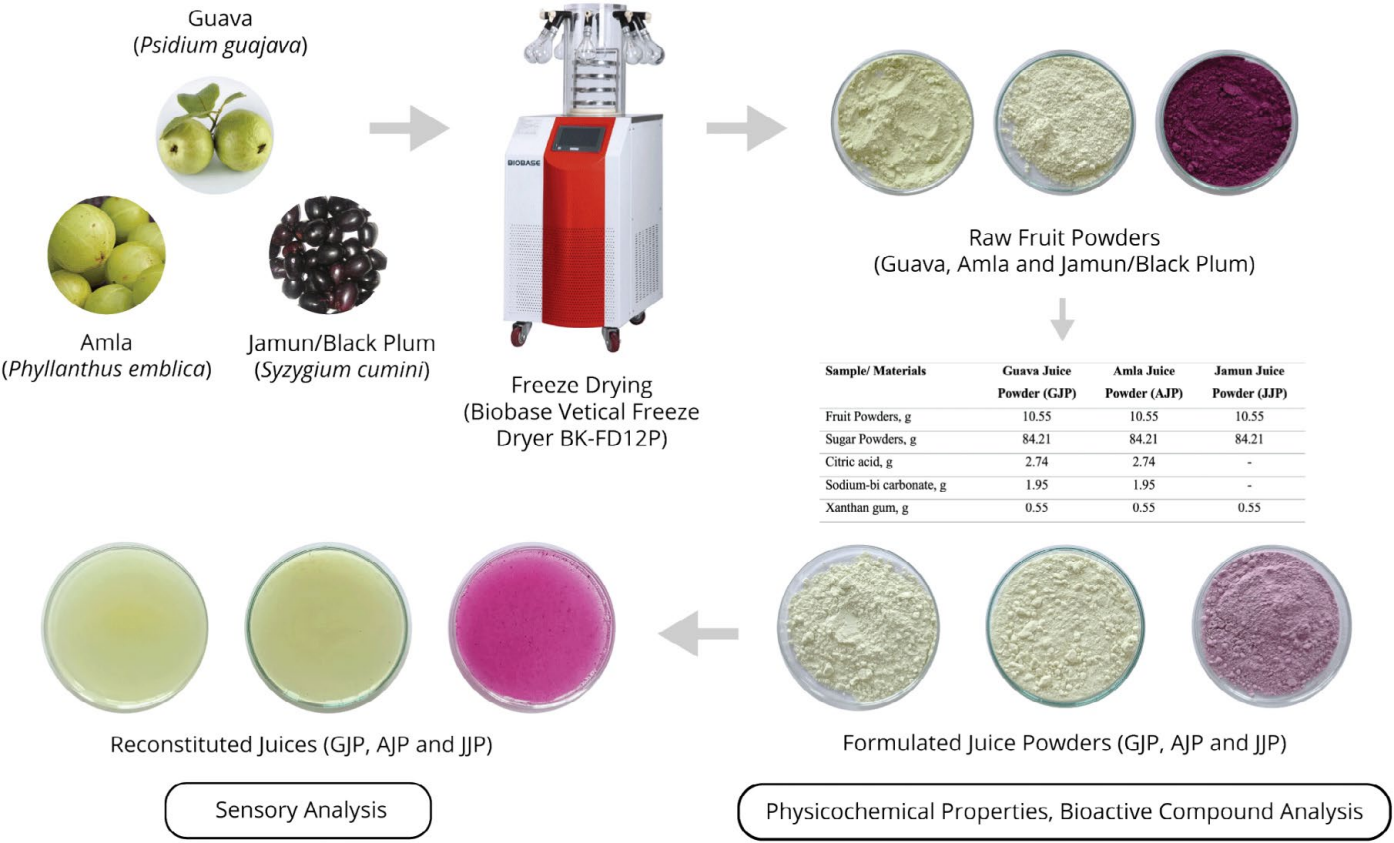
Overall approach of the study.

The current study was conducted in the Food Processing and Preservation Laboratory of Hajee Mohammad Danesh Science and Technology University, Dinajpur, Bangladesh. The overall approach of the study is depicted in Figure 1.

### 2.1 Chemical and Reagents

All chemicals and reagents required for different analyses, including metaphosphoric acid, sodium hydroxide (NaOH), sodium bicarbonate (NaHCO), Folin–Ciocalteu reagent (FCR), 2,2‐diphenyl‐1‐picrylhydrazyl (DPPH), sodium nitrite (NaNO_2_), aluminum chloride (AlCl_3_), n‐hexane, acetone, ethanol, methanol, phosphate buffer, acetate buffer, alpha‐glucosidase, PNPG (4‐nitrophenyl‐β‐D‐glucopyranoside), and potassium di‐hydrogen orthophosphate, citric acid, sodium bicarbonate, and xanthan gum were exclusively sourced from Sigma‐Aldrich, Germany, and were analytical grade. Sugar was obtained from a local grocery store in Dinajpur.

### 2.2 Raw Materials Processing

The key components of this investigation, specifically the fruits guava (average weight ~ 160 g, light green in color), amla (average weight ~ 15 g, light yellow), and jamun (average weight ~ 9 g, deep purple)—were sourced from the Bangladesh Agricultural Research Institute (BARI), located in Joydebpur, Gazipur, Dhaka, Bangladesh. The fruits were fresh, free from diseases, and commercially mature.

They underwent thorough cleaning with regular tap water to eliminate surface contaminants. Subsequently, the clean fruits were deseeded using a spoon and chopped into small pieces. In the case of guava, the fruits were sliced into small pieces first, and seeds were then removed using a knife, as the study used the “Thai hybrid variety,” which contains very few seeds.

The freeze‐drying method, as described by Islam and Hasan (2024), was employed with slight modifications to dry the fruit pieces. The fruit pieces were placed in trays within the freeze dryer (Biobase Vetical Freeze Dryer BK‐FD12P). A 15 mm layer of the samples was evenly distributed in each tray, with approximately 300 g of guava, 310 g of amla, and 320 g of jamun per tray. Initially, the trays were subjected to a freezing chamber at −60°C for 8 h. Following proper freezing, the vacuum pressure was set to 10 Pa, and drying was completed for 30 ± 6 h at (−50°C to 25°C).

The resulting dried fruits were carefully gathered and individually stored in airtight glass containers. The dried fruit samples were ground using an electric laboratory grinder and subsequently sieved through a mesh #50 screen to achieve a powder with a precise particle size of 300 μm. The resultant fruit powders were then carefully stored at −18°C, awaiting subsequent processing stages.

### 2.3 Formulation of Fruit Juice Powder

Through iterative trials (data are not shown), varying ingredient proportions, ready‐to‐drink juice powder samples were formulated using specific fruit powders. The final compositions of these formulations are detailed in Table 1.

**TABLE 1:** Formulation of 100 g of ready‐to‐drink fruit juice powder (except JJP).

Sample/materials	Guava juice powder (GJP)	Amla juice powder (AJP)	Jamun juice powder (JJP)
Fruit powders, g	10.55	10.55	10.55
Sugar powders, g	84.21	84.21	84.21
Citric acid, g	2.74	2.74	—
Sodium‐bi carbonate, g	1.95	1.95	—
Xanthan gum, g	0.55	0.55	0.55

**FIGURE 2:**
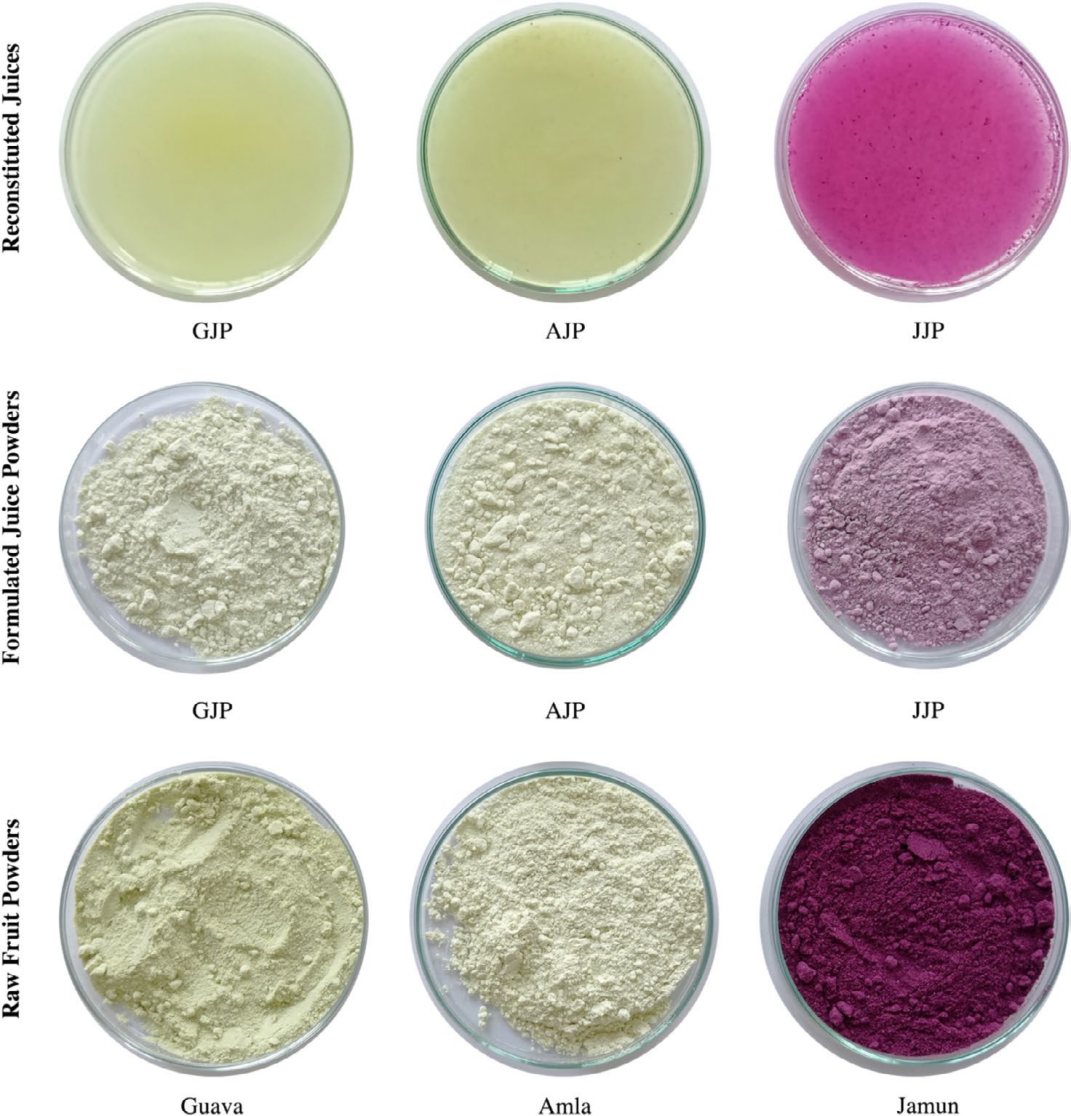
Raw fruit powders (freeze‐dried), formulated and reconstituted juice samples.

The juice powder (5 g) was reconstituted by adding water (200 mL) for sensory evaluation and color analysis. For other analyses, juices were prepared following established methodological protocols. Raw fruit powders, formulated and reconstituted juice samples are highlighted in Figure 2.

### 2.4 Extraction of Bioactive Compounds From Formulated Fruit Juice Powders

With a slight modification of the organic solvent extraction method outlined by Islam et al. (2021), the bioactive components of each fruit juice powder recipe (GJP, AJP, and JJP) were extracted. Initially, a solid/liquid ratio of 1:10 (v/w) was established by dissolving 2.5 g of the prepared powder in 50 mL of 80% methanol. Subsequently, the mixture was transferred to a magnetic stirrer and run at a speed of 100 rpm for an hour at room temperature. The mixtures were then centrifuged (MF 300, Hanil Science Industrial Co., Incheon, Korea) at 4000 rpm for 10 min. After that, using a 10 mL plastic syringe, the supernatant aliquot was transferred and filtered with a Whatman filter number one. The filtrates (extracts) were then stored for further analysis.

### 2.5 Determination of Physical Properties of Juice Powders 

#### 2.5.1 Color Evaluation

A digital colorimeter (BC‐110/200, Biobase, China) was used to measure the color parameters L (lightness), a (redness to bluishness), and b (yellowness to greenness) of the instant fruit juice powder and the reconstituted juice (5 g of powder samples were dissolved in 200 mL of water). The chroma and hue were calculated using the following equations adopted from Rahman et al. (2024):

(1) $$\text{Chroma},C=\sqrt{a^{2}+b^{2}}$$

(2) $$\mathrm{Hue}=\tan^{-1}\left(\frac{b}{a}\right)$$

#### 2.5.2 Bulk, Tapped, and Particle Density

The bulk density, tapped density, and particle density of the prepared juice powder formulation were determined following the methods outlined by Budnimath et al. (2023). Bulk density (weight per volume) was evaluated by weighing 1 g of fruit powder samples in a graduated 10 mL cylinder to measure the occupied volume. After tapping the cylinder 50 times in 5 min, the volume was remeasured to determine the tapped density. For particle density, 1 g of juice powder sample was placed into a 10 mL graduated cylinder with a stopper on top. Then, 5 mL of petroleum ether was added, and the mixture was shaken at 100 rpm to suspend all the particles. The cylinder wall was washed with 1 mL of petroleum ether, and the total volume was recorded (Demircan et al. 2023). The bulk density, tapped density, and particle density of the prepared juice powder formulation were determined following Equations ((3), (4), (5)):

(3) $$\text{Bulk desnity},\mathrm{BD}=\frac{\text{Weight of the juice powder},\;g}{\text{Volume},\;\mathrm{mL}}$$

(4) $$\text{Tapped desnity},\mathrm{TD}=\frac{\text{Weight of the juice powder},\;g}{\text{Volume after Tapping},\;\mathrm{mL}}$$

(5) $$\begin{array}{c} \text{Particle desnity},\mathrm{PD}=\\ \;\frac{\text{Weight of the juice powder},\;g}{\text{Total volume of petrolueam ether and juice}\;\text{powder},\;{\mathrm{mL}}^{-6}} \end{array}$$

#### 2.5.3 Flowability, Cohesiveness, and Porosity

The flowability and cohesiveness of the prepared juice powder formulation were determined in terms of Carr index and Hausner ratio using bulk density and tapped density values. The porosity was calculated from particle density and tapped density (Budnimath et al. 2023) using the following Equations ((6), (7), (8)):

(6) $$\text{Carr index},\mathrm{CI}=\frac{\mathrm{TD}-\mathrm{BD}}{\mathrm{TD}}\times 100$$

(7) $$\text{Hausner}\;s\;\text{ratio},\mathrm{HR}=\frac{\mathrm{TD}}{\mathrm{BD}}$$

(8) $$\text{Porosity}=\frac{\mathrm{PD}-\mathrm{TD}}{\mathrm{PD}}\times 100$$

Here BD is bulk density, TD is tapped density, and PD is particle density.

#### 2.5.4 Dispersibility and Wettability

Dispersibility of the powder formulations was measured following the method outlined by Jinapong et al. (2008). At room temperature, in a 50 mL beaker, 10 mL distilled water and 1 g juice powder sample were taken. Using a vortex mixer, the mixture was shaken vigorously for 15 s, and then the reconstituted powder was sieved through a 212 μm sieve. The separated powder was weighed and dried for 4 h in a hot air oven at 105°C to obtain the % dry matter. Dispersibility (%) was calculated using the following Equation (9):

(9) $$\text{Dispersibility}\;\left(\%\right)=\frac{\left(10+a\right)\times \%\mathrm{TS}}{a\times \left(100-b\right)/100}$$

Here, a is the amount of powder used, b is the moisture content in the powder, and %TS is the dry matter in the sieved powder.

The wettability was determined by the method outlined by Budnimath et al. (2023), where 1 g of juice powder sample was placed on the surface of 400 mL distilled water at room temperature. The stopwatch was used to record the time required for complete submersion of the juice powder.

#### 2.5.5 Water Solubility Index (WSI) and Water Absorption Index (WAI)

The water solubility index (WSI) and water absorption index (WAI) were measured, slightly modifying the method described by Acosta‐Vega et al. (2023) and Igual et al. (2021). In a centrifuge tube, 30 mL of water and 2.5 g of juice powders were taken, then stirred for 30 min, followed by centrifugation at 5100 rpm (MF 300, Hanil Science Industrial Co., Incheon, Korea) for 5 min. The supernatant was siphoned off to a petri dish. The separated supernatant and remaining sediment were dried in an oven overnight at 105°C. Equations (10) and (11) were used to calculate WSI and WAI:

(10) $$\begin{array}{cc} \text{Water solubility index},\mathrm{WSI}\;\left(\%\right)\;=\; & \frac{\text{Dried weight of the supernatant},\;g}{\text{Initial sample weight},\;g}\\ & \times 100 \end{array}$$

(11) $$\text{Water absorption index},\mathrm{WAI}=\frac{\text{Weight of the dried sediment},\;g}{\text{Initial sample weight},\;g}$$

#### 2.5.6 Solubility

The solubility of the juice powder was determined using a slightly modified method described by Naji‐Tabasi et al. (2021). One gram of the juice powder was added to 100 mL of distilled water and stirred for 5 min, followed by centrifugation at 5100 rpm for 10 min. An aliquot of 25 mL supernatant was weighed and oven‐dried for 5 h at 105°C. The solubility percentage was calculated from the difference in weight.

#### 2.5.7 Moisture Content

Using a halogen moisture analyzer (XY‐105 MW, Wincom, China), the moisture content (on a dry basis) was calculated (Dazon et al. 2019). The analyzer was validated with reference powder materials of known moisture contents. The sample tray, pan holder, tray holder, and wind cover were meticulously assembled following the provided instructions. The analyzer was then calibrated for sample weight. Afterward, approximately 2.0 g of the sample was placed on the tray, and upon completion of the analysis a few minutes later, the recorded moisture content was noted.

#### 2.5.8 Particle Size

Following the indirect method of particle size determination as illustrated by Barbosa‐Cánovas et al. (2009), the manual process involves sieving the powders through a stack of sieves arranged in ascending order of aperture size. The powder is initially placed on the top sieve, and the weight of particles retained on each sieve is recorded at the end of sieving. In this study, we used sieve no. 50, which is equivalent to 300 μm. Sieving continued until no oversize remains.

#### 2.5.9 Dissolution Time

The dissolution time of the formulated fruit juice powders was determined by slightly modifying the method outlined by Hadree et al. (2023). Five grams of the sample were added to 200 mL of water at room temperature and shaken using a vortex mixer. The time taken for complete dissolution, resulting in a homogeneous solution without visible residues, was recorded in seconds as the dissolution time.

#### 2.5.10 pH and Total Soluble Solids

First, 5 g of powder samples were dissolved in 200 mL of water. Then, the pH and total soluble solids (TSS), °Brix, were determined using a digital pH meter and a refractometer, respectively.

### 2.6 Chemical Analysis of the Juice Powders

#### 2.6.1 Vitamin C Content

Vitamin C was determined using a modified spectrophotometric method outlined by Rahman et al. (2024). One gram of dried material was placed in a sterile Falcon tube, and 10 mL of 1% metaphosphoric acid was used for extraction. The sample was allowed to sit at ambient temperature for 45 min in a dark environment, followed by filtration using Whatman filter no. 4 paper and a Hoover filter pump. The filtered sample was then stored in a dark location. Subsequently, 2.75 mL of 2,6‐dichlorophenolindophenol and 250 μL of the filtrated sample were combined in a 10 mL measuring cylinder. This mixture was left undisturbed for 25 min in a dark place. The absorbance at 515 nm was measured using a spectrophotometer (UV 1900i, Shimadzu, Japan) and estimated the vitamin C using ascorbic acid as standard.

#### 2.6.2 Total Carotenoid Content

The quantification of total carotenoids was conducted following the methodology outlined by Hasan, Kabir, et al. (2022). One gram of the sample was placed in a conical flask. A 50 mL solution was prepared with a 50:25:25 ratio of n‐hexane, acetone, and ethanol. This solution was combined with the sample in the conical flask, and the flask was sealed with aluminum foil. The mixture underwent ultrasonic (Model: JP‐010 T, Skymen Cleaning Equipment Shenzhen Co. Ltd., China) stimulation for 10 min, with intermittent shaking every 3 min to prevent heat interference. Subsequently, the mixture was centrifuged (MF‐300, HumanLab Instrument Co., Korea) for 10 min at 4000 rpm. The extraction solution was used to create a 50 mL volume from the supernatant, which was then collected in a falcon tube and stored in a dark place. Using β‐carotene as a standard, the absorbance was measured at 450 nm in a spectrophotometer (UV 1900i, Shimadzu, Japan), and the results were reported as μM β‐carotene equivalents per gram of dry sample.

#### 2.6.3 Total Phenolic Content (TPC)

Total phenolic content (TPC) was determined following the method of Islam et al. (2023). A standard curve for TPC was prepared using gallic acid (0–20 μM), and the results were reported as mg of gallic acid equivalent per 100 g (mg GAE/100 g) of the sample. In a 10 μL measuring cylinder, 500 μL of sample extract and 500 μL of Folin–Ciocalteu solution were combined. This mixture was then mixed with 1 mL of 7.5% NaHCO_3_ solution. Subsequently, 10 mL of distilled water was added to the mixture, and it was vortexed briefly. The mixture was allowed to sit at room temperature for 35 min in a dark place. Afterward, the supernatant was collected, and proper blanks were used for background subtraction. The absorbance at 750 nm was measured using a spectrophotometer (UV 1900i, Shimadzu, Japan).

#### 2.6.4 Total Flavonoid Content (TFC)

With a slight modification, the colorimetric approach reported by Islam et al. (2021) was utilized to determine the total flavonoid content (TFC). TFC was determined using a standard quercetin calibration curve (0–300 μM), and the results were expressed as mg of quercetin equivalents per 100 g (mg QE/100 g) of the sample. A 0.3 mL of 5% NaNO_2_ was added to 1 mL of extract dissolved in 4 mL of distilled water in a centrifuge tube. After allowing the tubes to stand for 5 min, 0.3 mL of 10% AlCl_3_ was added to the mixture, and it was left to stand for an additional minute. Finally, 2.4 mL of distilled water and 2 mL of 1 M NaOH were added, and the mixture was vortexed immediately. The tubes were then centrifuged for 10 min at 4000 rpm and left at room temperature for 15 min in a dark location. The absorbance at 510 nm was measured using a spectrophotometer (UV‐1800, Shimadzu Corporation, Japan), compared to a blank prepared in a similar way but with methanol instead of the extract.

#### 2.6.5 Antioxidant Activity (DPPH Assay)

The antioxidant properties of the fruit powder were evaluated using DPPH antioxidant assays. First, the 2,2‐diphenyl‐1‐picrylhydrazyl (DPPH) scavenging capacity assay was conducted following the protocol outlined by Hasan, Islam, et al. (2022). Using a spectrophotometer (UV‐19001, Shimadzu, Japan), the absorbance of a DPPH solution in 80% methanol was adjusted to 0.650 at 515 nm after 30 min of stirring. Following that, 1.950 mL of DPPH was added to 50 μL of the extracted sample, which was vortexed and kept at room temperature for 30 min in the dark. The mixture's absorbance was then measured at 515 nm, and the results were expressed as μM Trolox equivalents per gram of dry matter (μM Trolox/g DM), calculated using a standard Trolox curve. The DPPH scavenging activity was calculated using the following Equation (12):

(12) $$\text{DPPH scavenging ability}\;\left(\%\right)=\frac{A\;\text{control}-A\;\text{sample}}{\text{Acontrol}}\times 100$$

where A represents the absorbance at 515 nm.

### 2.7 Sensory Evaluation

With the assistance of 31 semi‐trained panelists (17 male, 14 female), including academicians and students aged between 21 and 35 years, the sensory evaluation of the reconstituted juice (prepared by dissolving 5 g of powder sample in 200 mL of water) samples was conducted. Panelists received a thorough briefing before the evaluation, and verbal agreement was obtained. The evaluation was conducted in a well‐lit, quiet room at ambient temperature. Coded samples were served in randomized order, and panelists used drinking water for palate cleansing between samples. The assessment was conducted using a 9‐point hedonic scale (1 = Dislike extremely to 9 = Like extremely). Panelists were instructed to score the samples on appearance, flavor, taste, mouthfeel, solubility, and overall acceptability (Kabir et al. 2024).

### 2.8 Statistical Analysis

All analyses were conducted in three replicates, and the results are presented as the mean ± standard deviation. One‐way analysis of variance (ANOVA) and Duncan's multiple range test (DMRT) were performed using IBM SPSS Statistics version 25 at a 95% significance level.

## 3 Results and Discussion

### 3.1 Physicochemical Properties of the Juice Powder

#### 3.1.1 Color Evaluation

This study assessed the color properties of Guava Juice Powder (GJP), Amla Juice Powder (AJP), and Jamun Juice Powder (JJP), revealing significant differences in L* values between powder and reconstituted forms (Table 2). The high L* values in powders, GJP at 93.89, AJP at 90.38, and JJP at 82.97, indicate a bright appearance, with GJP being the lightest. Upon reconstitution, L* values dropped to approximately 35 across all samples, suggesting a darker, possibly richer hue that might influence consumer perception of quality. This shift aligns with findings by Gomes et al. (2018) and Kha et al. (2010), who observed similar reductions in L* values after reconstitution during spray‐drying of papaya pulp and Gac fruit powder. This could be attributed to rehydration, which reintroduces water and leads to darker hues due to pigment reactivation and increased light absorption (Gomes et al. 2018). As fruit juices are consumed mostly for their functional attributes, such shifts in color might not significantly impact consumer perception. However, further research is needed to evaluate whether such changes are critical in industrial processes where color consistency is essential.

**TABLE 2:** Color evaluation of juice powders and reconstituted juices.

Samples	L*	a*	b*	Chroma	Hue
Raw juice powder					
GJP	93.89 ± 0.95^a^	−3.74 ± 0.35^b^	10.63 ± 0.54^a^	11.27 ± 0.60^a^	−1.232 ± 0.045^b^
AJP	90.38 ± 1.47^b^	−4.85 ± 1.17^b^	11.12 ± 1.32^a^	12.14 ± 1.67^a^	−1.159 ± 0.015^b^
JJP	82.97 ± 1.06^c^	6.37 ± 0.27^a^	0.11 ± 0.20^b^	6.37 ± 0.27^b^	0.017 ± 0.026^a^
Reconstituted juice					
GJP	35.08 ± 2.18^a^	0.96 ± 0.10^b^	2.34 ± 0.31^a^	2.53 ± 0.25^a^	1.18 ± 0.08^a^
AJP	35.98 ± 0.59^a^	1.03 ± 0.04^b^	2.31 ± 0.17^a^	2.53 ± 0.14^a^	1.15 ± 0.04^a^
JJP	34.62 ± 0.28^a^	1.34 ± 0.02^a^	1.95 ± 0.04^a^	2.37 ± 0.03^a^	0.97 ± 0.02^b^

*Note:* Each value was measured in triplicates and expressed as mean ± SD, and different small letters within the same column indicate significant (*p* ≤ 0.05) differences among the samples.

Our analysis of a* values, representing the red‐green spectrum, showed distinct color profiles for GJP, AJP, and JJP in both powder and reconstituted forms. In powder form, GJP and AJP exhibited negative a* values (−3.74 and − 4.85, respectively), suggesting a slight green tint, while JJP had a positive a* value (6.37), indicating a red hue, most likely due to its natural pigments. Upon reconstitution, all samples exhibited positive a* values, with JJP showing the highest (1.34), suggesting a shift towards redness in the liquid form. This shift is consistent with previous studies, where reconstitution led to changes in a* values due to alterations in pigment dispersion and light scattering (Gomes et al. 2018).

We also evaluated the b* values, which represent the yellow‐blue spectrum. These values were positive across all samples, indicating a yellowish hue. GJP and AJP powders had higher b* values (10.63 and 11.12, respectively), showing strong yellowish tones, while JJP had a low b* value (0.11), indicating minimal yellowness, consistent with its darker appearance. After reconstitution, b* values decreased across all samples, with JJP at 1.95 showing the least yellowness. This suggests that dilution reduces the yellow component, potentially affecting perceived vibrancy. These findings are in agreement with Kha et al. (2010), who reported reduced yellowness in reconstituted spray‐dried fruit powders due to water‐induced pigment changes. The high b* in GJP and AJP powders could appeal to consumers seeking bright, tropical tones, while JJP's lower b* aligns with consumer preferences for darker juices.

We assessed chroma, a measure of color saturation, across GJP, AJP, and JJP, and observed notable differences between powder and reconstituted forms. In powder form, GJP (11.27) and AJP (12.14) exhibited high chroma, indicating vivid colors, whereas JJP's lower chroma (6.37) reflected its muted saturation, consistent with its darker tone. Upon reconstitution, chroma decreased to around 2.5 in all samples, suggesting less intense colors in the liquid form, which may affect perceived freshness. This reduction in chroma after reconstitution aligns with previous findings, where dilution led to decreased color intensity in fruit powders (Lee et al. 2017).

We analyzed hue angles to understand color shifts in GJP, AJP, and JJP across forms. In powder form, GJP (−1.232) and AJP (−1.159) had negative hue angles, placing them in the fourth quadrant (greenish‐yellow), while JJP's near‐zero hue (0.017) positioned it at the red‐yellow boundary, reflecting its distinct pigmentation. Reconstitution shifted all hues to positive values, GJP (1.18), AJP (1.15), and JJP (0.97) moving them into the red‐yellow quadrant, suggesting warmer tones in the liquid form. Such hue shifts upon reconstitution have been reported in other fruit juice powders and are attributed to changes in pigment interactions and light‐scattering behavior in the liquid matrix (Herbach et al. 2007).

Our controlled drying conditions may have enhanced color stability compared to industrial settings. Further studies are warranted to refine the understanding of processing impacts on juice color and consumer perception.

#### 3.1.2 Moisture Content

This study measured the moisture content in Guava Juice Powder (GJP), Amla Juice Powder (AJP), and Jamun Juice Powder (JJP) to assess storage stability, a key factor in food product shelf life. Moisture levels were found to be 2.36%, 1.77%, and 2.21% for GJP, AJP, and JJP, respectively (Table 3), indicating low water activity that reduces the risk of microbial spoilage, as lower moisture content limits microbial growth (Akhter et al. 2024). These values align closely with (Naji‐Tabasi et al. 2021) Naji‐Tabasi et al. (2021), who found similar moisture levels (around 2%) in berry juice powders, suggesting that our freeze‐drying process achieves stability comparable to established methods.

**TABLE 3:** Physicochemical properties of juice powder samples.

Parameters	Guava juice powder (GJP)	Amla juice powder (AJP)	Jamun juice powder (JJP)
Moisture content (%)	2.36 ± 0.06^a^	1.77 ± 0.04^c^	2.21 ± 0.05^b^
pH	5.60 ± 0.01^b^	5.40 ± 0.01^c^	6.60 ± 0.01^a^
Total soluble solids (TSS), Brix	9.00 ± 0.01^a^	8.20 ± 0.01^c^	8.50 ± 0.02^b^
Bulk density (g/mL)	0.38 ± 0.03^a^	0.39 ± 0.05^a^	0.42 ± 0.06^a^
Tapped density (g/mL)	0.56 ± 0.02^b^	0.50 ± 0.03^c^	0.67 ± 0.02^a^
Particle density (g/mL)	4.76 ± 0.45^a^	4.35 ± 0.25^b^	5.26 ± 0.13^a^
Flowability (Carr index)	30.81 ± 1.50^b^	23.20 ± 0.70^c^	37.54 ± 0.34^a^
Cohesiveness (Hausner ratio)	1.45 ± 0.07^ab^	1.30 ± 0.10^b^	1.60 ± 0.20^a^
Porosity (%)	88.34 ± 1.09^a^	88.50 ± 0.30^a^	87.35 ± 0.75^a^
Solubility (%)	41.30 ± 0.50^b^	39.11 ± 0.64^c^	49.00 ± 0.60^a^
Wettability (s)	104.00 ± 3.00^a^	97.00 ± 2.00^b^	50.00 ± 2.00^c^
Dispersibility (%)	50.54 ± 0.39^b^	54.50 ± 0.10^a^	41.80 ± 0.55^c^
Water solubility index (%)	33.52 ± 0.84^c^	56.37 ± 0.53^a^	42.47 ± 0.8^b^
Water absorption index	5.09 ± 0.07^b^	4.40 ± 0.17^c^	8.93 ± 0.15^a^

*Note:* Each value was measured in triplicates and expressed as mean ± SD, and different small letters within the same row indicate significant (*p* ≤ 0.05) differences among the samples.

Among the three, AJP showed the lowest moisture content, implying the highest potential for extended shelf life. Although the moisture contents of GJP and JJP were slightly higher, they still remained well below the 5% threshold, which is generally considered safe for dried foods in terms of microbial stability (Fellows 2022). This provides a competitive advantage for these juice powders in terms of shelf life. However, due to the hygroscopic nature of powdered foods, packaging with high barrier properties is essential to maintain quality. Moreover, while our lab‐scale drying was unaffected by external environmental conditions such as humidity and temperature, these factors could influence moisture content under industrial settings. This highlights the need for further research under real‐world conditions. In conclusion, the low moisture content of the instant fruit juice powders indicates good storage stability and strong potential for commercialization and extended shelf life.

#### 3.1.3 pH and Total Soluble Solids (TSS)

We studied the pH and total soluble solids (TSS) in reconstituted guava juice powder (GJP), amla juice powder (AJP), and jamun juice powder (JJP) to evaluate acidity and sweetness, which are critical for taste, flavor, and preservation. The pH values of the juice samples were recorded as 5.60, 5.40, and 6.60 for GJP, AJP, and JJP, respectively (Table 3), indicating mildly acidic conditions typical of fruit juices. However, these values are slightly higher than anticipated, despite the addition of citric acid in GJP and AJP. This could be attributed to the reaction between sodium bicarbonate and citric acid, which produces carbon dioxide (CO_2_) effervescence upon reconstitution with water. The reaction leads to partial neutralization of citric acid, thereby slightly increasing the pH. Similar observations were reported by Madhava et al. (2020) for reconstituted pineapple juice.

The TSS levels were measured at 9.00, 8.20, and 8.50 °Brix for GJP, AJP, and JJP, respectively (Table 3), suggesting consistent sweetness across formulations despite variations in ingredients. These values are comparable to those reported by Rather et al. (2024), who observed a TSS range of 7.22–8.50 °Brix in different formulations of soy‐whey‐fortified pineapple juice. The TSS of reconstituted juices may vary depending on the quantity and type of ingredients used in the juice formulation. Overall, the slightly acidic pH contributes to microbial stability, while appropriate TSS levels enhance palatability.

#### 3.1.4 Bulk, Tapped and Particle Density

This study evaluated the bulk, tapped, and particle densities of GJP, AJP, and JJP to assess their physical properties, which are important for storage, packaging volume, and transportation efficiency and cost (Vivek, Mishra, et al. 2020). Bulk density, reflecting the powder volume in a loose state, was recorded as 0.38, 0.39, and 0.42 g/mL for GJP, AJP, and JJP, respectively (Table 3), closely matching the values reported for freeze‐dried orange juice powder (0.32–0.37 g/mL) by Uscanga et al. (2021).

Tapped density, measured after mechanical compaction, was higher, 0.56, 0.50, and 0.67 g/mL for GJP, AJP, and JJP respectively, exceeding the range reported by Uscanga et al. (2021) for orange juice powder (0.39–0.46 g/mL). The higher tapped densities may be attributed to the larger particle sizes of the powders, possibly due to their high pulp content (Shenoy et al. 2015).

Particle density, which reflects the true density of the solid material, was 4.76, 4.35, and 5.26 g/mL for GJP, AJP, and JJP, respectively, aligning with Demircan et al. (2023), who reported a particle density of 5.00 g/mL for orange juice powder. However, this study did not evaluate the particle size distribution of the freeze‐dried fruit powders and the reconstituted juices. Further research is recommended to investigate particle agglomeration, moisture absorption, and density variations under uniform particle size conditions.

#### 3.1.5 Flowability, Cohesiveness, and Porosity

Both consumers and manufacturers value the free‐flowing nature of food powders, as it facilitates accurate dosing, improves industrial processing (such as mixing and packaging), and enhances ease of handling for consumers. Therefore, flowability and cohesiveness are crucial physical properties of food powders. This study assessed the flowability, cohesiveness, and porosity of GJP, AJP, and JJP to evaluate their handling and reconstitution characteristics, essential for both consumer satisfaction and industrial performance.

Flowability, measured by the Carr index (CI), indicates compressibility, where lower values indicate better flow characteristics. In this study, the CI values were 30.81 for GJP, 23.20 for AJP, and 37.54 for JJP (Table 3). These results suggest that AJP possessed fair flowability, while GJP exhibited poor flow, and JJP demonstrated very poor flow. Similarly, cohesiveness, indicated by the Hausner ratio (HR), showed values of 1.45 (GJP), 1.30 (AJP), and 1.60 (JJP). According to classification criteria by Naji‐Tabasi et al. (2021), HR values above 1.25 suggest increasing cohesiveness and poorer flow, which aligns with our CI results. Interestingly, these CI and HR ranges are comparable to those reported by Shelke et al. (2023), who reported 20.89–35.90 for spray‐dried jamun juice powder, suggesting our freeze‐dried powders exhibit similar flow behavior despite different drying methods. This consistency highlights the inherent difficulty of achieving good flow in powders derived from high‐pulp or sugar‐rich fruits like the three fruits we used. Further supporting this, a recent study on freeze‐dried bacuri pulp powder (a fruit known for high viscosity and sugar content) reported CI values of 23.21%–25.80% and HR of 1.30–1.34 even when using carrier agents like maltodextrin or gum arabic (Moura et al. 2024). This suggests that the fair‐to‐poor flowability observed in our GJP, AJP, and JJP, produced without such agents, is characteristic of freeze‐dried powders derived directly from fruit matrices. The high pulp and sugar content likely contribute to inter‐particle interactions, increasing cohesiveness and hindering flow. Moisture content also plays a critical role, as residual water can form liquid bridges between particles, increasing cohesiveness (İzli et al. 2022). Microencapsulation is commonly used to improve powder flow properties (Sandhya et al. 2025).

Porosity, which influences bulk density and reconstitution behavior, is another critical parameter. Higher porosity generally enhances water penetration during rehydration (Hajiaghaei and Sharifi 2022). The measured porosities were high and relatively similar across the samples: 88.34% (GJP), 88.50% (AJP), and 87.35% (JJP) (Table 3). These values are noted to be consistent with spray‐dried orange juice powder but slightly higher than freeze‐dried orange juice powder reported previously (Stavra et al. 2022). While high porosity is generally favorable for rehydration, the actual reconstitution behavior also depends on other factors like particle size, surface characteristics, and composition.

#### 3.1.6 Dispersibility and Wettability

Reconstitution behavior is a key aspect influencing consumer acceptance of instant juice powders. Among the critical parameters are dispersibility—the ability of powder clumps to break down into individual particles upon contact with water—and wettability—the time required for the powder to become fully submerged when placed on the water's surface. These are essential quality criteria for instant juice powders and are influenced by composition, surface chemistry, particle size distribution, and particle density (Ding et al. 2020; Felix da Silva et al. 2020). Together, these factors determine the degree of “instantness” of the product.

The dispersibility measured for the powders was 50.54% for GJP, 54.50% for AJP, and 41.80% for JJP (Table 3). These values indicate moderate dispersibility. While specific classifications can vary, this range suggests that a reasonable portion of the powder separates into finer particles upon mixing. These results are lower than those reported for some freeze‐dried orange juice powders but comparable to spray‐dried versions (Stavra et al. 2022), potentially reflecting differences in particle structure or composition inherent to the fruits or drying nuances. The variation between the samples (AJP being highest, JJP lowest) may relate to differences in their insoluble solids content or particle surface characteristics affecting aggregation.

Wettability, measured as the time (in seconds) for complete submersion, was 104 s (GJP), 97 s (AJP), and 50 s (JJP) (Table 3). Following standard interpretations (e.g., > 120 s = poor, 61–120 s = moderate, < 60s = good) (Wang et al. 2023), JJP demonstrated good wettability, while GJP and AJP showed moderate wettability. The faster wetting of JJP is advantageous for instant properties. This contrasts somewhat with its lower dispersibility and could be linked to specific surface properties or perhaps its slightly different porosity (Section 3.1.5) facilitating initial water penetration, even if aggregate breakdown is slower (Fournaise et al. 2021). The moderate wettability times for GJP and AJP are still acceptable for many applications and fall within ranges reported for other freeze‐dried fruit powders like mulberry (Wang et al. 2023). Overall, the good‐to‐moderate wettability suggests the powders interact readily with water, a crucial factor for consumer convenience in juice preparation.

#### 3.1.7 Water Solubility Index (WSI) and Water Absorption Index (WAI)

The Water Solubility Index (WSI) quantifies the proportion of soluble solids released from a powder into water, reflecting its dissolution potential, an attribute often influenced by processing parameters (İzli et al. 2022). Conversely, the Water Absorption Index (WAI) measures the amount of water absorbed and retained by the powder's insoluble components upon reconstitution. Both parameters are important indicators of how a powder interacts with water (Igual et al. 2023).

In this study, the WSI values were 33.52% for GJP, 56.37% for AJP, and 42.47% for JJP (Table 3). High WSI is generally desirable for instant juice powders to ensure rapid and complete dissolution. While acceptable values depend on the product type, a WSI above 50% is recommended for instant juice powders (Ouyang et al. 2024).

Among the samples, AJP exceeded this benchmark, indicating excellent solubility characteristics. JJP approached the desired level, while GJP fell slightly below, suggesting lower solubility. The relatively high WSI of AJP (56.37%) may be attributed to its composition, possibly containing higher levels of soluble acids or sugars. JJP exhibited moderate solubility (42.47%), and GJP showed lower solubility (33.52%), which may indicate a greater proportion of insoluble constituents.

The WAI values were 5.09 g/g for GJP, 4.40 g/g for AJP, and 8.93 g/g for JJP (Table 3), reflecting the water‐holding capacity of their insoluble matrices. The values for GJP and AJP are broadly comparable to those reported by Anisuzzaman et al. (2023) for spray‐dried tomato powder. The WAI of a powder may vary depending on the intended use. For instant juice powders aiming for a thin consistency, lower WAI is often preferred alongside high WSI. However, a higher WAI might be acceptable or even desirable if a thicker mouthfeel is intended. The high WAI of JJP could potentially lead to a thicker reconstituted juice compared to GJP and AJP (Mirzazadeh et al. 2024).

#### 3.1.8 Solubility

The solubility of juice powder is another important rehydration property, as it relates to consumer preferences and serves as a reliable measure for understanding powder behavior in water (Naji‐Tabasi et al. 2021; Zotarelli et al. 2022). In this study, the solubility values were 41.30% for GJP, 39.11% for AJP, and 49.00% for JJP (Table 3), with JJP exhibiting the highest dissolution efficiency. These values are comparable to those reported by Si et al. (2016) for freeze‐dried raspberry powder but are lower than those observed by Naji‐Tabasi et al. (2021) for spray‐dried barberry juice powder. The relatively lower solubility observed in the present study may be attributed to the freeze‐drying process, which tends to preserve the fiber fraction of the fruit pulp without significant structural breakdown, resulting in reduced solubility. Furthermore, the absence of carrier or coating agents such as maltodextrin, commonly used in spray drying to enhance solubility, could have contributed to the lower solubility (Caparino et al. 2012). This highlights a known trade‐off of freeze‐drying: superior nutrient retention at the cost of lower reconstitution performance. Among the samples, JJP's higher solubility suggests it may offer better consumer acceptability, while the lower solubility of GJP and AJP reflects limitations associated with freeze‐drying unmodified fruit matrices. These findings underscore the potential benefit of formulation optimization, such as incorporating carrier agents, to improve the solubility and overall functionality of freeze‐dried juice powders.

#### 3.1.9 Particle Size Distribution

This study analyzed the particle size distribution of GJP, AJP, and JJP to understand its impact on functional properties like solubility, wettability, flowability, and viscosity of reconstituted juice (Gawałek 2021; Zotarelli et al. 2022). All samples exhibited particle sizes under 300 μm, with 100% passing through a No. 50 sieve (Table 3), classifying them as coarse powders, similar to freeze‐dried orange juice powder studied by Camacho et al. (2023). Unlike spray‐drying, which uses carriers like maltodextrin to produce finer particles (Zotarelli et al. 2022), our freeze‐drying without carriers resulted in larger, coarser particles, potentially adjustable with further grinding.

### 3.2 Bioactive Compound and Functional Properties of Juice Powder

#### 3.2.1 Vitamin C Content

Vitamin C is an antioxidant and is associated with various immune functions (Kamrul et al. 2016) and the ability to fight infections. Vitamin C deficiency can lead to a significant decline in health. The vitamin C content of the prepared fruit juice powder was determined to be 116.21, 154.49, and 176.89 μM ascorbic acid/g DM, respectively, for GJP, AJP, and JJP (Table 4). This result is comparable to that observed by Rohini et al. (2024) for lemon juice powder formulation. Considering the proportions used in the recipe, the vitamin C content of the guava formulation is comparable to that reported by Verma et al. (2015) for freeze‐dried guava powder. For amla, our findings were lower compared to Mishra et al. (2009) but considerably higher than what was found by Tewari et al. (2021). The vitamin C content of jamun was found to be higher than that reported by Jebitta et al. (2016). The use of low temperatures in freeze‐drying typically contributes to the effective retention of ascorbic acid; however, it is well known that the variety and growing conditions of fruits significantly affect their vitamin C content. Results from our study show that each of the juices contains a good amount of vitamin C, which can contribute to daily vitamin C intake.

**TABLE 4:** Bioactive compound and functional properties of juice powder.

Parameters	Guava juice powder (GJP)	Amla juice powder (AJP)	Jamun juices powder (JJP)
Vitamin C (μM ascorbic acid/g DM)	116.21 ± 1.62^c^	154.49 ± 13.14^b^	176.89 ± 4.90^a^
Total carotenoid (μM β‐carotene/g)	7.97 ± 0.28^a^	8.03 ± 0.24^a^	5.09 ± 0.02^b^
Total phenolic content (mg GAE/100 g)	280.56 ± 10.67^c^	822.62 ± 7.42^a^	333.13 ± 11.93^b^
Total flavonoid content (mg QE/100 g)	98.21 ± 9.45^c^	607.74 ± 8.25^a^	377.98 ± 65.11^b^
Antioxidant activity (μM Trolox/g DM)	43.05 ± 0.38^b^	45.88 ± 0.24^a^	45.90 ± 0.08^a^

*Note:* Each value was measured in triplicates and expressed as mean ± SD and different small letters within the same row indicate significant (*p* ≤ 0.05) differences among the samples.

#### 3.2.2 Total Carotenoid Content

Carotenoids are known for their antioxidant and anti‐inflammatory activities and are associated with reducing chronic health issues, maintaining healthy blood concentrations, and offering numerous other health benefits (Hasan et al. 2024). They are also provitamin A compounds, contributing to approximately 30% of the vitamin A intake in the human diet (Bohn et al. 2021). The total carotenoid content was 7.97, 8.03, and 5.09 μM β‐carotene equivalent for GJP, AJP, and JJP, respectively (Table 4). Although none of these fruits are renowned for their carotenoid profiles, the juice powders showed comparable carotenoid content to commercial orange juices (Sánchez‐Moreno et al. 2006) and Cape gooseberry (Ordóñez‐Santos et al. 2017). The content was higher than that found in commercial cashew apple products Assunção and Mercadante (2003) but lower than in some commercial milk‐fruit juices (Stinco et al. 2019). According to the results, the prepared juice will certainly contribute to a healthy lifestyle for the consumers.

#### 3.2.3 Total Phenolic Content (TPC)

Phenolic compounds in food are known for their beneficial effects on health. They contribute to protecting against cardiovascular diseases, cancer, metabolic disorders, neurodegenerative diseases, and autoimmune conditions (Dias et al. 2021). The TPC of the prepared juice powder recipes was determined to be 280.56, 822.62, and 333.13 mg GAE/100 g for GJP, AJP, and JJP, respectively (Table 4). These values are comparable to the findings of Brzezowska et al. (2023), who prepared beverage powders from various vegetable and fruit mixes and reported TPC content in a similar range. Hajiaghaei and Sharifi (2022) reported slightly higher TPC values for instant juice powder made from quince fruit using foam mat drying. The drying method often affects TPC content, with thermal hydrolysis during high‐temperature drying potentially leading to an increase in phenolic compounds. Based on these results, the prepared fruit powder juices are likely to provide health benefits due to their significant TPC content.

#### 3.2.4 Total Flavonoid Content (TFC)

Flavonoids, known for their anti‐inflammatory, antimicrobial, and antioxidant properties, help reduce the risk of various diseases and contribute to numerous health benefits (Shen et al. 2022). The TFC content of GJP, AJP, and JJP was found to be 98.21, 607.74, and 377.98 mg QE/100 g, respectively (Table 4). The results are comparable to the study conducted by Correa Uriburu et al. (2023) for spray‐dried beverage powder from two Argentine native plants and Hamid et al. (2020), where they studied freeze‐dried juices powder prepared from wild pomegranate. The results of TFC content in this study prove the potential of the prepared juices to provide health benefits through their flavonoid content.

#### 3.2.5 Antioxidant Activity (DPPH Assays)

Antioxidants protect cells from damage caused by oxidative stress from free radicals (Hasan et al. 2019). The antioxidant activity of a sample is often correlated with its phenolic and flavonoid compounds content (Hasan, Islam, et al. 2022). Using the DPPH assay, the antioxidant activity was measured as 43.05, 45.88, and 45.90 μM Trolox equivalents/g DM for GJP, AJP, and JJP, respectively (Table 4). These values were higher than those reported by de Tavares et al. (2020) for jambolana powder using foam mat drying, and significantly lower than the values found by Can‐Cauich et al. (2017) for freeze‐dried fruit peel. These variations can be attributed to differences in fruit varieties, recipe formulations, and the drying methods employed. It is well established that freeze drying tends to retain higher antioxidant activity compared to other drying methods (Hamid, Thakur, Thakur, et al. 2020). The fruit powder juice samples prepared from all three fruits showed an ample amount of antioxidative activity, which should benefit consumers by providing protection against aging and offer other health benefits associated with antioxidants.

### 3.3 Sensory Evaluation

**FIGURE 3:**
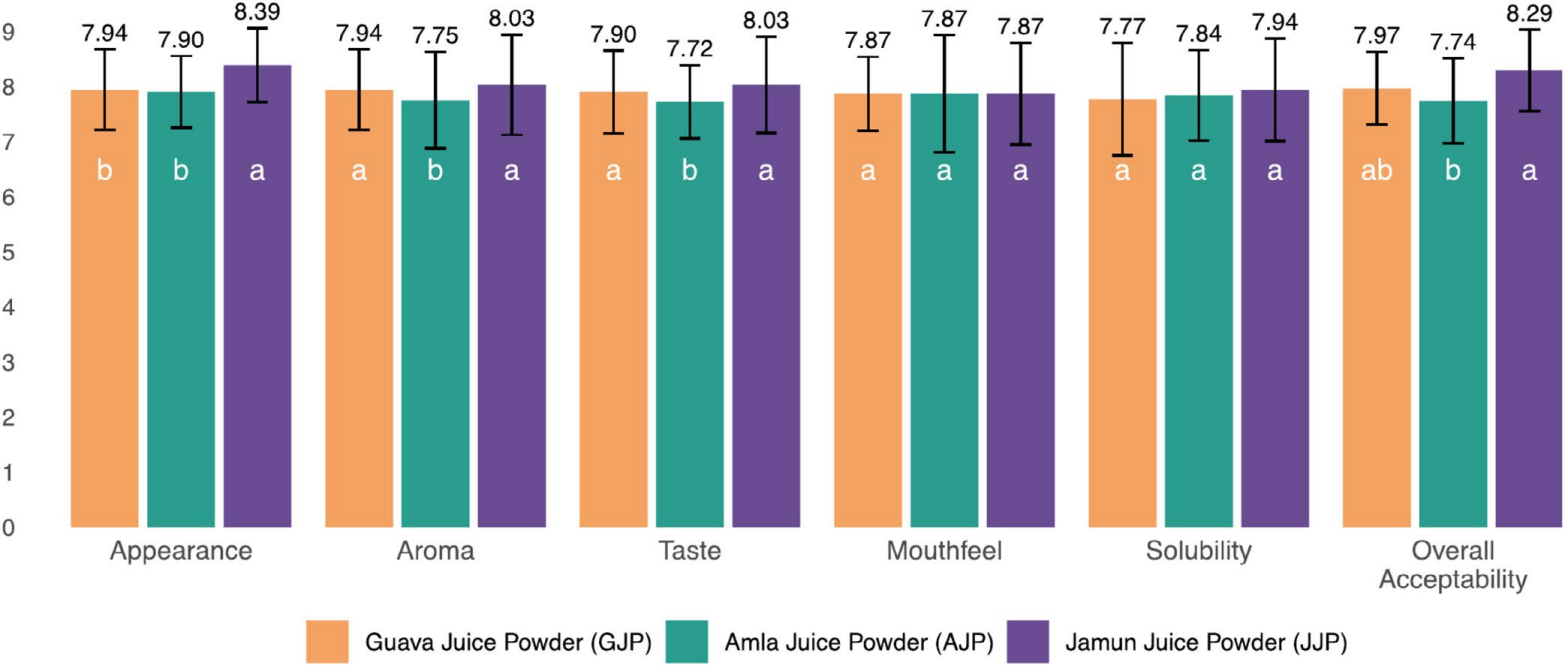
Sensory evaluation scores of reconstituted juices prepared from GJP, AJP, and JJP (*n* = 31). Error bars represent the standard deviations. Different lowercase letters on the bars indicate statistically significant differences (*p* < 0.05) among the juice samples according to Duncan's multiple range test (DMRT).

Sensory evaluation is essential for assessing and understanding consumer acceptance of a novel food product. It helps evaluate consumers' sensory responses to the new product (Vivek, Subbarao, et al. 2020). The sensory evaluation results of our formulated juice powders indicated that the reconstituted juices prepared using the JJP recipe received the highest scores across all metrics (Figure 3). Despite the absence of an effervescent agent in the JJP formulation, it demonstrated superior performance in terms of appearance, aroma, taste, and solubility, followed by GJP and AJP. Appearance is crucial as it creates the first impression and influences consumer preference. A visually appealing product often reflects high quality (Kabir et al. 2024). The appearance scores for GJP, AJP, and JJP were 7.94, 7.90, and 8.39, respectively; JJP achieved the highest appearance score, indicating a more visually appealing product. This difference can be attributed to the distinct and vibrant color of JJP (Figure 1 and Table 2). Although GJP and AJP were more flowable and less cohesive compared to JJP, the color of JJP helped it stand out.

Aroma contributes to the naturalness and authenticity of the juice, which is important for health‐conscious consumers (Pan et al. 2023). Therefore, retaining the natural aroma of fruit is essential. The aroma ratings were 7.94 for GJP, 7.75 for AJP, and 8.03 for JJP. JJP was rated highest for aroma upon reconstitution. The absence of additional agents like citric acid and sodium bicarbonate allowed the aroma to be more prominent in JJP compared to the other formulations. The scores of the taste parameter were 7.90, 7.72, and 8.03 for GJP, AJP, and JJP, respectively. Although there is no statistically significant difference, JJP scored higher than the other two. This difference may be attributed to its formulation, which lacks effervescent agents.

In terms of mouthfeel, all three samples scored the same, 7.87, indicating consistent texture across all samples. Despite variations in other sensory attributes, the mouthfeel was rated similarly for all formulations.

The solubility ratings were 7.77 for GJP, 7.84 for AJP, and 7.94 for JJP. The JJP demonstrated better solubility, which can be attributed to the absence of sodium bicarbonate and citric acid in its formulation.

The appearance, taste, aroma, mouthfeel, and solubility collectively influenced the overall acceptability scores. The scores were 7.97 for GJP, 7.74 for AJP, and 8.29 for JJP. The JJP achieved the highest overall acceptability score, attributed to its superior appearance, taste, aroma, and solubility. This indicates a strong preference for JJP among the panelists, with AJP being the least preferred and GJP falling in between. Statistical analysis supported these findings. Although GJP and AJP scored slightly lower than JJP, they still received high ratings, approaching a hedonic scale of 8, which corresponds to ‘like very much.’ Therefore, it can be concluded that all three juice samples are sufficiently attractive to consumers in the instant juice powder segment.

## 4 Conclusions

This study successfully developed freeze‐dried instant juice powders from guava (GJP), amla (AJP), and jamun (JJP), achieving the objective of enhancing the nutritional value and shelf stability of tropical fruits. Freeze‐drying effectively preserved the sensory qualities and bioactive compounds, resulting in powders rich in vitamin C, phenolics, and flavonoids, with strong antioxidant activity. The powders also exhibited favorable physical and functional properties, supporting ease of handling, solubility, and consumer acceptability. Among the formulations, JJP received the highest overall sensory score, indicating its strong consumer appeal. These results support the feasibility of freeze‐drying as a viable preservation technique for developing functional juice powders. Although freeze‐drying is generally considered more expensive than methods such as spray drying, its superior quality and nutrient retention may justify the investment. Future research should focus on process optimization, cost–benefit analysis, and the inclusion of other underutilized fruits to broaden product development opportunities.

## Author Contributions

**Md. Rafiquzzaman:** data curation (equal), formal analysis (equal), investigation (equal), methodology (equal), writing – original draft (equal). **Md Akram Hossain:** formal analysis (equal), methodology (equal), software (equal), supervision (equal), validation (equal), visualization (equal), writing – original draft (equal), writing – review and editing (equal). **Rifat Rafique Dwip:** data curation (equal), formal analysis (equal), investigation (equal), methodology (equal), writing – original draft (equal). **Musfika Jahan:** data curation (equal), formal analysis (equal), investigation (equal), methodology (equal), writing – original draft (equal). **S. M. Kamrul Hasan:** conceptualization (equal), methodology (equal), resources (equal), software (equal), supervision (equal), validation (equal), visualization (equal), writing – review and editing (equal).

## Ethics Statement

All relevant rules, guidelines, and regulations were followed, and consent was sought and obtained from all panelists/participants for sensory analysis in this study.

## Conflicts of Interest

The authors declare no conflicts of interest.

## Data Availability

The data that support the findings of this study are available from the corresponding author upon reasonable request.
